# Phenotypic Stability of Energy Balance Responses to Experimental Total Sleep Deprivation and Sleep Restriction in Healthy Adults

**DOI:** 10.3390/nu8120823

**Published:** 2016-12-19

**Authors:** Laura E. Dennis, Andrea M. Spaeth, Namni Goel

**Affiliations:** 1 Division of Sleep and Chronobiology, Department of Psychiatry, Perelman School of Medicine at the University of Pennsylvania, Philadelphia, PA 19104, USA; ledennis@mail.med.upenn.edu; 2 Center for Obesity Research and Education, College of Public Health, Temple University, Philadelphia, PA 19122, USA; andrea.spaeth@temple.edu

**Keywords:** individual differences, sleep restriction, total sleep deprivation, recovery, caloric intake, late-night intake, macronutrients, gender differences

## Abstract

Experimental studies have shown that sleep restriction (SR) and total sleep deprivation (TSD) produce increased caloric intake, greater fat consumption, and increased late-night eating. However, whether individuals show similar energy intake responses to both SR and TSD remains unknown. A total of *N* = 66 healthy adults (aged 21–50 years, 48.5% women, 72.7% African American) participated in a within-subjects laboratory protocol to compare daily and late-night intake between one night of SR (4 h time in bed, 04:00–08:00) and one night of TSD (0 h time in bed) conditions. We also examined intake responses during subsequent recovery from SR or TSD and investigated gender differences. Caloric and macronutrient intake during the day following SR and TSD were moderately to substantially consistent within individuals (Intraclass Correlation Coefficients: 0.34–0.75). During the late-night period of SR (22:00–04:00) and TSD (22:00–06:00), such consistency was slight to moderate, and participants consumed a greater percentage of calories from protein (*p* = 0.01) and saturated fat (*p* = 0.02) during SR, despite comparable caloric intake (*p* = 0.12). Similarly, participants consumed a greater percentage of calories from saturated fat during the day following SR than TSD (*p* = 0.03). Participants also consumed a greater percentage of calories from protein during recovery after TSD (*p* < 0.001). Caloric intake was greater in men during late-night hours and the day following sleep loss. This is the first evidence of phenotypic trait-like stability and differential vulnerability of energy balance responses to two commonly experienced types of sleep loss: our findings open the door for biomarker discovery and countermeasure development to predict and mitigate this critical health-related vulnerability.

## 1. Introduction

Experimental studies have demonstrated causal mechanisms between short sleep duration and obesity risk. In healthy adults, sleep restriction (curtailed sleep across multiple consecutive days) leads to increases in caloric intake [1,2,3,4,5,6,7,8,9,10], snacking [7,11], fat and carbohydrate consumption [2,5,7,8,10,11], late-night eating/delayed meal timing [1,2,10] and weight gain [1,2,3]. Similarly, during total sleep deprivation (one night of continuous wakefulness), adults consume a large number of calories during the overnight period [12], consume more fat the following day [12], make more food purchases [13], consume larger portion sizes [14] and eat more calories from snacks [14].

This increased consumption of energy (via food/drink) [9] exceeds the additional energy required to sustain the extended wakefulness associated with either type of sleep loss [1,15,16,17]. Notably, the additional energy cost differs by sleep loss type: approximately 100 additional calories are required during sleep restriction [1,15], while 135 additional calories are required during total sleep deprivation [16], suggesting that intake amounts may differ during and following these sleep loss types to compensate for differential increases in energy expenditure.

Studies have found neurobehavioral vulnerability to sleep loss shows individual differences and is trait-like and stable within individuals across sleep restriction and total sleep deprivation [18,19]. Previously, we observed individual differences in the increased caloric intake, late-night eating and weight gain responses to sleep restriction but stability in the responses within individuals during two sleep restriction exposures separated by long time intervals [20]. However, it remains unknown whether energy balance responses are also trait-like and stable within individuals across different types of sleep loss, namely sleep restriction and total sleep deprivation. 

Separate studies of sleep restriction [1,2,3,21,22] and total sleep deprivation [16] have demonstrated energy balance responses to sleep loss return to baseline levels after one or more nights of recovery sleep. Whether this recovery and its time course is similar following sleep restriction and total sleep deprivation remains unknown. Although the time course for recovery for some objective and subjective measures of sleepiness and cognitive performance from sleep restriction and total sleep deprivation is similar [23,24,25,26,27,28,29,30], this is not true for all tests [30,31]. Thus, it is possible that recovery of energy balance measures may differ depending on the type of sleep loss experienced.

Gender differences also have been observed in the energy balance response to sleep loss. During sleep restriction, men exhibit lower subjective ratings of fullness [5] and greater increases in caloric intake [1,10,21], consume more calories during late-night hours [10], and gain more weight than women [1,2]. Furthermore, these energy balance responses are more stable across repeated exposures to sleep restriction in men than women [20]. Notably, evidence for gender differences in objective energy balance responses during total sleep deprivation is limited since the majority of such studies either have used only men or used sample sizes precluding reliable gender comparisons [32]. Thus, additional research is needed to identify gender differences in energy balance responses during total sleep deprivation, and moreover, to compare such responses to those observed during sleep restriction.

In the current study, we examined daily and late-night caloric and macronutrient intake responses to one night of sleep restriction and one night of total sleep deprivation, as well as intake responses during subsequent recovery from both types of sleep loss. Given the stability of neurobehavioral responses across sleep restriction and total sleep deprivation and the stability of energy balance responses across two separate sleep restriction exposures, we hypothesized intake responses would be consistent across one night of sleep restriction and one night of total sleep deprivation. We also hypothesized recovery intake responses would be similar after sleep restriction or total sleep deprivation, given prior neurobehavioral response findings. Finally, we predicted men would exhibit greater late-night and caloric intake responses to both sleep restriction and total sleep deprivation, given prior reports of gender differences in energy intake.

## 2. Materials and Methods 

### 2.1. Participants

Healthy individuals between the ages of 21 and 50 years old were recruited in response to study advertisements. Participants reported habitual nightly sleep durations between 6.5 h and 8.5 h, with habitual bedtimes between 22:00 and 00:00 and habitual awakenings between 06:00 and 09:30; these were confirmed via wrist actigraphy. Chronotype was determined via the Morningness–Eveningness Composite Scale [33]. Participants did not engage in habitual napping and did not present with sleep disturbances (i.e., no complaints of daytime sleepiness, insomnia, or other sleep–wake disturbances). They did not have any acute or chronic psychological and medical conditions, as determined by questionnaires, interviews, physical exams, clinical history, and urine and blood tests (including a fasting blood glucose test). They were not taking any regular medications (except oral contraceptives) and were nonsmokers with body mass indices (BMIs) between 17.3 and 30.3 kg/m^2^. They did not participate in transmeridian travel or shift work, or have irregular sleep–wake routines in the 60 days before the study. Participants were monitored at home with actigraphy, sleep–wake diaries, and time-stamped call-ins to determine bedtimes and waketimes during the 7–14 days before the laboratory phase and the 7 days following the laboratory phase. Sleep disorders were excluded on the first laboratory night by oximetry and polysomnography measurements. Participants were not allowed to use tobacco during the 7 days before the study, as verified by blood and urine screenings. The protocol was approved by the University of Pennsylvania’s Institutional Review Board (IRB number: 812523). All participants provided written informed consent in accordance with the Declaration of Helsinki. They received compensation for participation.

### 2.2. Procedure

Participants engaged in a 13-day laboratory study in which they were studied continuously, and received daily checks of vital signs and symptoms by nurses (with a physician on call). All participants experienced two types of sleep loss during the protocol—sleep restriction (SR) and total sleep deprivation (TSD)—with the order of sleep loss exposures counterbalanced across conditions. Participants were randomized as a group (*N* = 4 per group) to one of the two conditions after two initial nights of baseline sleep (BL1-2) of 10 h (22:00–08:00) and 12 h (22:00–10:00) time in bed (TIB) respectively, and were blinded to condition assignment until after the second night of baseline sleep. Participants randomized to Condition A (*N* = 34) underwent five consecutive nights of sleep restricted to 4 h TIB per night (SR1-5, 04:00–08:00) followed by four consecutive nights of 12 h recovery sleep (R1–R4, 22:00–10:00), one night of total sleep deprivation (TSD, 0 h TIB) during which they were kept awake for 36 h (10:00–22:00 the following day), and then a final night of recovery sleep (R5, 22:00–10:00). Participants randomized to Condition B (*N* = 32) underwent one night of total sleep deprivation (TSD, 0 h TIB) during which they were kept awake for 36 h (10:00–22:00 the following day), followed by four consecutive nights of 12 h recovery sleep (R1–R4, 22:00–10:00), five consecutive nights of sleep restricted to 4 h TIB per night (SR1-5, 04:00–08:00) and then a final night of recovery sleep (R5, 22:00–10:00). Participants were discharged from the study on the day following R5.

Participants were ambulatory and were permitted to perform sedentary activities such as watching television, reading, and playing video or board games between cognitive test bouts (completed while seated at a computer); however, they were not allowed to exercise. Ambient temperature was maintained between 22 °C and 24 °C. Laboratory light levels remained constant at <50 lux during scheduled wakefulness and <1 lux during scheduled sleep periods. Participants were monitored continuously by trained staff throughout the study to ensure adherence.

### 2.3. Measures

Participants chose their meals/snacks from various menu options, and selected additional food/drink (including chips, cookies, fruit, low-fat yogurt, caffeine-free soda and juices) available in the laboratory kitchen, which included an industrial-size refrigerator, microwave, and toaster oven. They could also make requests to the study coordinator and study monitors. To ensure participants had sufficient time to eat each day, three 30 to 45-min meal opportunities were specified in the study during days with a 22:00 bedtime and one additional 30-min meal opportunity was specified at 00:45 during SR and TSD. Beyond these allotted meal times, participants could also consume food/drink at any time during the study during wakefulness except when they were performing cognitive tests. Participants were not told they must eat/drink and they were instructed to eat/drink whenever they desired as long as doing so did not interfere with cognitive testing. Furthermore, participants could eat the items they had ordered or could select from other foods available in the laboratory kitchen, and could eat as much or as little as they desired. Participants retrieved their own food/drink from the laboratory kitchen when they wanted to eat/drink and had the choice of eating at a table in the common area or privately in their bedrooms. Participants were not permitted to consume caffeinated beverages or chocolate during the protocol.

All food was weighed and recorded before being given to the participants. Food items were served in individual containers to increase the measurement accuracy of each item’s weight. Each day, trained monitors recorded a detailed description, the amount consumed and the intake time of the items. In addition, any left-over food/drink after each meal was weighed and recorded. The intake data were entered into The Food Processor SQL program (version 10.11; ESHA Research, Salem, OR, USA), a validated [34] professional nutrition analysis software and database program that generates food/drink intake components including calories and macronutrients.

### 2.4. Statistical Analyses 

Mixed-model ANOVAs evaluated condition and sleep loss exposure type effects for late-night intake during the first night of SR and during TSD (SR: 22:00–04:00; TSD: 22:00–06:00) as well as for daily intake following the first night of SR (SR1: 08:00–22:00) and following TSD (06:00–22:00) for calories, macronutrients, saturated fat, sugar, and fiber. Mixed-model ANOVAs assessed gender and sleep loss exposure type effects for both late-night intake during and daily intake following sleep loss. 

Between-subjects ANOVAs, covarying baseline intake, compared intake on the day following recovery sleep from either SR or TSD (R1, recovery sleep between sleep loss exposures) for each intake variable. Mixed-model ANOVAs, covarying baseline intake, compared the time course of intake across the four recovery days (R1–R4) following consecutive recovery sleep nights from either SR or TSD for each intake variable. Intraclass correlation coefficients (ICCs) examined consistency in intake responses between SR and TSD. The following ranges characterize ICCs and reflect the stability of interindividual differences: 0.0–0.2 (slight); 0.2–0.4 (fair); 0.4–0.6 (moderate); 0.6–0.8 (substantial); and 0.8–1.0 (almost perfect) [35]. Statistical analyses were conducted using IBM SPSS Statistics for Windows (version 21).

## 3. Results

### 3.1. Participant Characteristics

Sixty-six participants (aged 21–50 years, 72.7% African American; 48.5% female) participated in the study, with *N* = 34 randomly assigned to Condition A (experienced five consecutive nights of SR first) and *N* = 32 randomly assigned to Condition B (experienced one night of TSD first). There were no significant differences between conditions in age (*p* = 0.28), BMI (*p* = 0.60), the percentage of participants who were African American (*p* = 0.69) or women (*p* = 0.81), or in chronotype (*p* = 0.07), pre-study sleep duration (*p* = 0.74) or midpoint (*p* = 0.26) (Table 1). Participants also did not differ significantly in caloric, macronutrient (protein, carbohydrate, fat), fiber, sugar or saturated fat intake (*p*’s > 0.08) during the first baseline day (08:00–22:00), which occurred prior to randomization.

### 3.2. Late-Night Intake during Sleep Restriction and Total Sleep Deprivation

**Figure 1:**
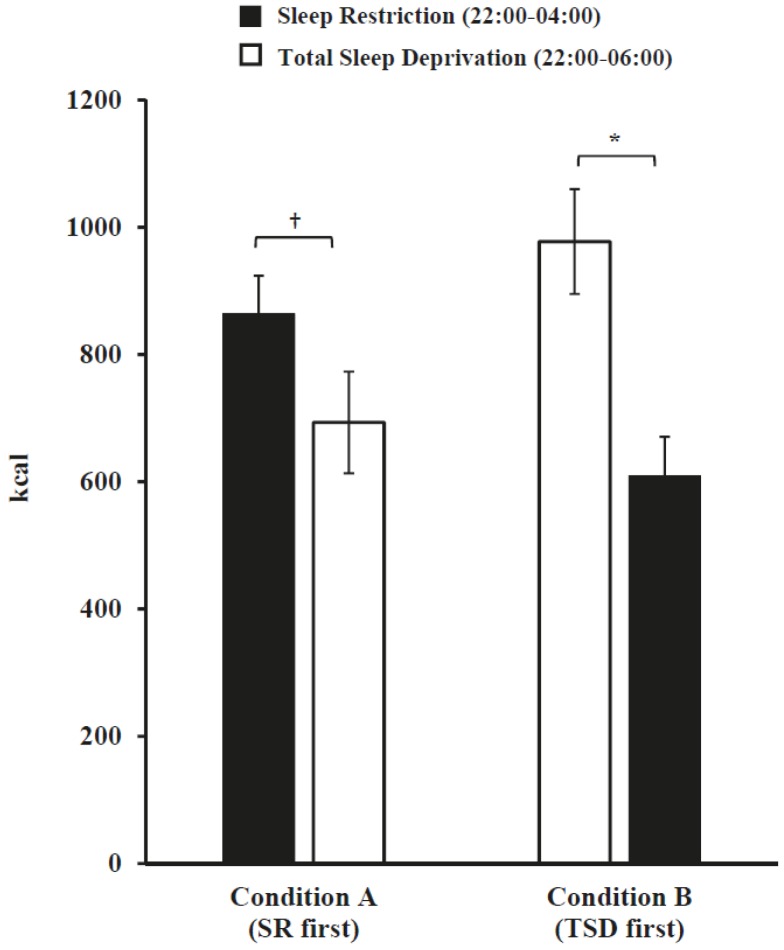
Mean ± SEM late-night caloric intake during sleep restriction (SR) and total sleep deprivation (TSD). There was a significant sleep loss exposure type (SR and TSD) × condition (A and B) interaction (*p* < 0.001), but no main effect of condition (*p* = 0.85). In both conditions, participants consumed more late-night calories during their first sleep loss exposure; however, this was statistically significant for Condition B (* *p* < 0.001), but not for Condition A († *p* = 0.08).

**Figure 2:**
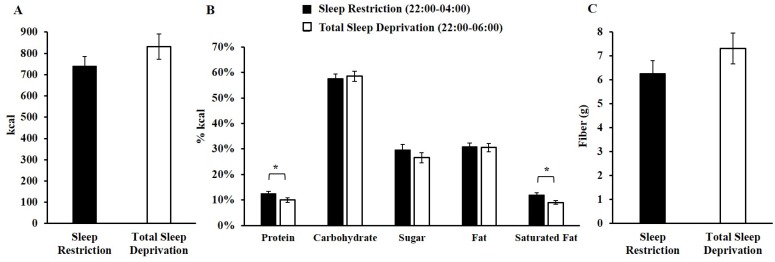
Mean ± SEM late-night intake during sleep restriction (SR) and total sleep deprivation (TSD). (**A**) Late-night caloric intake during SR and TSD did not significantly differ (*p* = 0.12); (**B**) During late-night hours, participants consumed a significantly larger percentage of calories from protein (* *p* = 0.01) and saturated fat (* *p* = 0.02) during SR, but there were no differences in carbohydrate, sugar, fat, or fiber (**C**) intake (*p*’s > 0.17).

For late-night caloric intake, there was a significant sleep loss exposure (SR and TSD) × condition (A and B) interaction (*F*(1, 64) = 18.05, *p* < 0.001) but no main effect of sleep exposure type (*p* = 0.12) or condition (*p* = 0.85). In both conditions, participants consumed more late-night calories during their first sleep loss exposure than during their second (Figure 1); however, this was only statistically significant for participants in Condition B (*F*(1, 31) = 18.49, *p* < 0.001) and not for those in Condition A (*F*(1, 33) = 3.37, *p* = 0.08). When examining late-night macronutrient, sugar, saturated fat and fiber intake, there were no significant sleep loss exposure × condition interactions (*p*’s > 0.11) or condition main effects (*p*’s > 0.20). There were significant sleep loss exposure main effects for protein and saturated fat: participants consumed a significantly larger percentage of calories from protein (*F*(1, 64) = 6.79, *p* = 0.01) and saturated fat (*F*(1, 64) = 5.79, *p* = 0.02) during SR late-night hours than during TSD late-night hours. By contrast, there were no significant sleep loss exposure main effects for caloric, carbohydrate, sugar, fat, or fiber intake (*p*’s > 0.17) (Figure 2).

For late-night caloric intake, while there was no significant sleep loss exposure (SR and TSD) × gender interaction effect (*p* = 0.24), there was a significant main effect of gender (*F*(1, 64) = 6.40, *p* = 0.01), whereby men consumed more late-night calories than women during both types of sleep loss (men: 878.4 ± 369.5 kcal, women: 687.5 ± 220.3 kcal). There were no significant sleep loss exposure (SR and TSD) × gender interactions (*p*’s > 0.07) or gender main effects (*p*’s > 0.19) for late-night macronutrient, sugar, saturated fat or fiber intake. 

ICC analyses, which examined the consistency in the late-night intake response between SR and TSD, ranged from slight to moderate: caloric intake: 0.18 (women: −0.42; men: 0.28); carbohydrate (%kcal): 0.18 (women: 0.31; men: 0.08); fat (%kcal): 0.03 (women: 0.14; men: −0.15); protein (%kcal): 0.55 (women: 0.59; men: 0.51); sugar (%kcal): 0.16 (women: 0.34; men: −0.06); saturated fat (%kcal): −0.25 (women: −0.90; men: −0.01); and fiber (g): 0.21 (women: 0.25; men: 0.19).

### 3.3. Daily Intake Following Sleep Restriction and Total Sleep Deprivation

**Figure 3:**
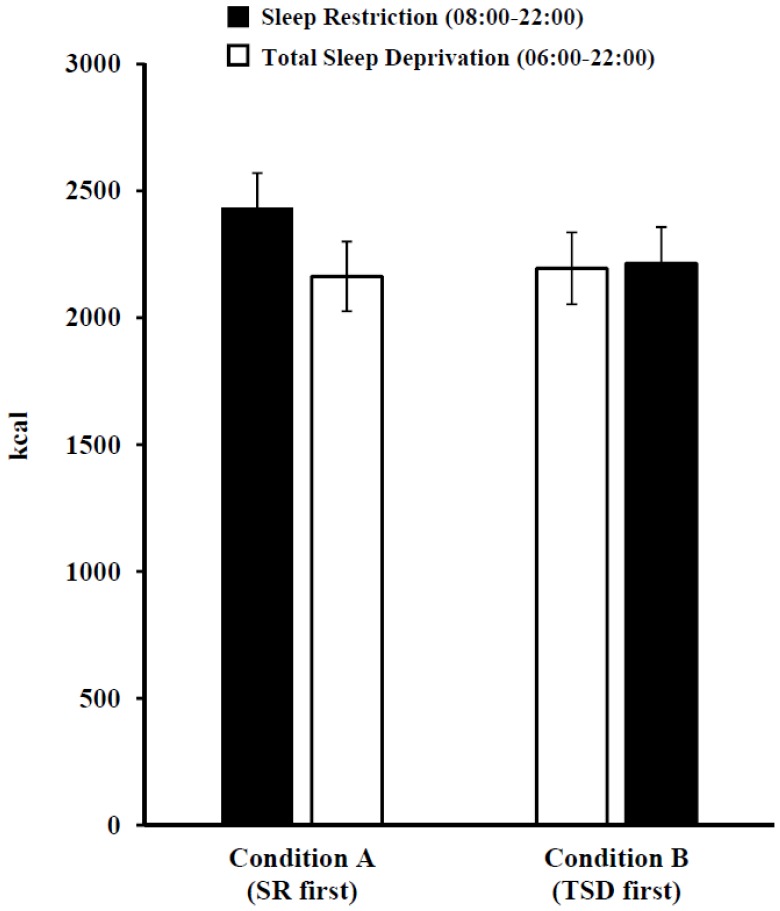
Mean ± SEM daily caloric intake following one night of sleep restriction (SR) and one night of total sleep deprivation (TSD). There was no significant sleep loss exposure type (SR and TSD) × condition (A and B) interaction effect (*p* = 0.16), and no significant main effects of sleep loss exposure type (*p* = 0.11) or condition (*p* = 0.61).

For protein intake, there was a significant main effect of condition (*F*(1, 64) = 4.83, *p* = 0.03): participants in Condition A consumed a greater percentage of calories from protein during the day following SR and TSD compared to participants in Condition B (A: 13.3%, B: 11.9%). However, there was no significant sleep loss exposure × condition interaction effect (*p* = 0.33) or main effect of sleep loss exposure type (*p* = 0.58). In addition, for saturated fat, there was a significant main effect of sleep loss exposure (*F*(1, 64) = 5.09, *p* = 0.03): participants consumed a greater percentage of calories from saturated fat during the day following SR than TSD (*p* = 0.03; Table 2). However, there was no significant sleep loss exposure × condition interaction effect (*p* = 0.09) or main effect of condition (*p* = 0.11). There were no significant sleep loss exposure (SR and TSD) × condition (A and B) interaction effects (*p*’s > 0.16) or main effects of sleep loss exposure type (*p*’s > 0.11, Table 2) or condition (*p*’s > 0.11) for caloric (Figure 3), carbohydrate, fat, sugar or fiber intake. 

While there were no significant sleep loss exposure (SR and TSD) × gender interactions for any intake variables (*p*’s > 0.12), there were main effects of gender for caloric intake (*F*(1, 64) = 14.09, *p* < 0.001) and protein intake (*F*(1, 64) = 5.02, *p* = 0.03). Compared to women, men consumed more calories and a greater percentage of calories from protein during the day following SR and TSD (Mean ± SD; men: 2546.4 ± 802.2 kcal, 13.3% ± 2.7% protein; women: 1940.8 ± 448.0 kcal, 11.9% ± 2.4% protein). There were no significant main effects of gender for carbohydrate, fat, sugar, saturated fat, or fiber intake (*p*’s > 0.22). 

**Figure 4:**
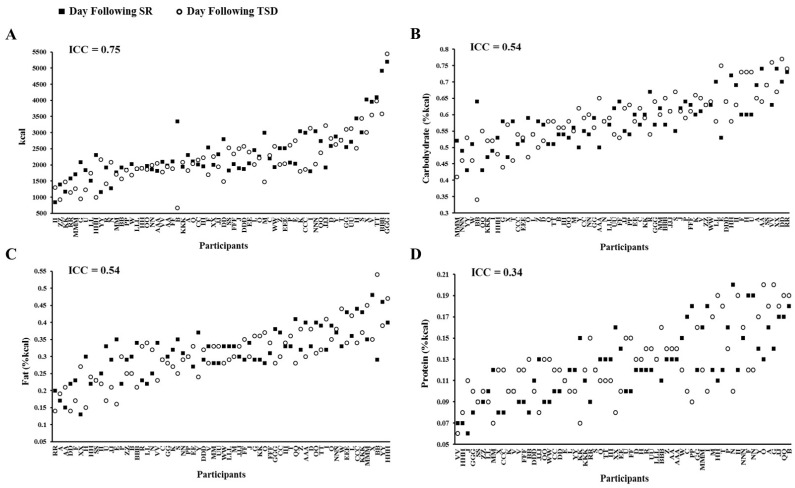
Individual differences and substantial phenotypic stability of caloric and macronutrient intake measures to sleep restriction (SR) and total sleep deprivation (TSD). Stability of caloric and macronutrient intake during the day following SR (08:00–22:00) and TSD (06:00–22:00) for (**A**) total caloric intake; (**B**) percentage of caloric intake from carbohydrates; (**C**) percentage of caloric intake from fat; and (**D**) percentage of caloric intake from protein. Participants (denoted individually with letters) are plotted in ascending order based on the mean intake of both sleep loss exposures (SR and TSD). See text for ICC ranges.

ICC analyses, which examined the consistency in daily intake following SR and TSD, ranged from fair to substantial (Figure 4): caloric intake: 0.75 (women: 0.54; men: 0.74); carbohydrate (%kcal): 0.54 (women: 0.45; men: 0.64); fat (%kcal): 0.54 (women: 0.54; men: 0.57); protein (%kcal): 0.34 (women: 0.24; men: 0.34); sugar (%kcal): 0.70 (women: 0.61; men: 0.77); saturated fat (%kcal): 0.59 (women: 0.52; men: 0.66); and fiber (g): 0.65 (women: 0.62; men: 0.67). 

### 3.4. Recovery Sleep Following Sleep Restriction or Total Sleep Deprivation

Because some participants received an Ensure nutrition shake during a metabolic testing procedure on the first recovery day, recovery data analyses were conducted only in the subset of participants who did not receive this nutrition shake (*N* = 24; Condition A: *N* = 12, Condition B: *N* = 12). In this subset, there were no significant differences between conditions in age (*p* = 0.15), BMI (*p* = 0.49), or in the percentage of participants who were African American (Condition A: 58.3%, Condition B: 41.7%; *p* = 0.51) or women (Condition A: 50.0%, Condition B: 50.0%, *p* = 1.0). Participants in each condition also did not differ in chronotype (*p* = 0.08) or pre-study sleep duration (*p* = 0.86) or midpoint (*p* = 0.14). 

Between-subjects ANOVAs, covarying baseline intake for each variable, compared intake during the day following the first recovery sleep night from either SR or TSD. Participants on the first day of recovery from TSD consumed a greater percentage of calories from protein than those on the first day of recovery from SR (*F*(1, 21) = 32.64, *p* < 0.001, Table 3). Participants on the first day of recovery from SR tended to consume more calories than those on the first day of recovery from TSD; however, this did not reach statistical significance (*F*(1, 21) = 3.80, *p* = 0.07, Table 3). There were no differences between conditions in carbohydrate, fat, saturated fat, sugar or fiber intake (*p*’s > 0.08).

Mixed-model ANOVAs, covarying baseline intake for each measure, compared the time course of intake across four recovery days following consecutive recovery nights (R1–R4, 10:00–22:00) from either SR or TSD. There were no recovery day main effects (*p*’s > 0.10) or recovery day × condition interaction effects for any intake measure (*p*’s > 0.06). There were no main effects of condition for daily caloric, carbohydrate, fat, saturated fat, sugar, or fiber intake (*p*’s > 0.11); however, participants across the four days of recovery from TSD consumed a greater percentage of calories from protein than those across the four days of recovery from SR (*F*(1, 21) = 19.00, *p* < 0.001).

## 4. Discussion

In this study, we found caloric and macronutrient intake during the day following one night of SR and one night of TSD were moderately to substantially consistent within individuals and showed differential vulnerability between individuals. During the late-night period of one night of SR and one night of TSD, consistency was slight to moderate; furthermore, although caloric intake did not differ, participants consumed a greater percentage of calories from protein and saturated fat during SR. Similarly, participants also consumed a greater percentage of calories from saturated fat during the day following SR than following TSD, despite comparable caloric intake. Notably, participants consumed a greater percentage of calories from protein during recovery after TSD. Caloric intake was greater in men during late-night hours and the day following sleep loss. These findings highlight the critical relationship between energy balance and sleep, and underscore the need to identify biomarkers and countermeasures to predict and mitigate energy balance vulnerability to sleep loss. Our findings are of timely importance, given recent, marked escalations in nighttime and overall caloric consumption, and the increased prevalence of both obesity and sleep loss in the population.

Caloric and macronutrient intake during the day following one night of SR and one night of TSD showed moderate to substantial consistency within individuals and differential vulnerability between individuals. Thus, if an individual was vulnerable to increased caloric and macronutrient intake in one type of sleep loss, he/she was also remarkably vulnerable to this eating behavior in the other type of sleep loss. These findings are in line with our prior study of the stability of energy balance responses across two separate sleep restriction exposures [20], and with studies showing stability of neurobehavioral responses across SR and TSD [18,19]. Future studies should examine the stability and individual differences of responses for another key component of energy balance—energy expenditure—as this measure differs between SR and TSD [1,15,16].

During the late-night period of one night of SR and one night of TSD, consistency was slight to moderate. These findings are in agreement with our prior study examining the stability of late-night responses across two separate sleep restriction exposures [20], which used comparable late-night eating intervals. Interestingly, although caloric intake did not differ, participants consumed a greater percentage of calories from protein and saturated fat during the late-night hours of SR than TSD, despite similar time intervals for eating. Participants also consumed a greater percentage of calories from saturated fat during the day following SR than following TSD, even though caloric intake was comparable. Our findings are consistent with other research showing increased protein and saturated fat intake during the SR period [36]. These macronutrient differences may be due to minor variations in late-night or daytime food selection and consumption, which could lead to differences in salt intake or other factors, by participants during SR and TSD.

Of interest, participants consumed more late-night calories during their first sleep loss exposure. There are several possible explanations for this finding. Participants may have overindulged in the first session due to the initial novelty of available food selections from the hospital menu and from the laboratory kitchen. In addition, the recovery sleep between sleep loss periods (12 h TIB for four nights) may have served as a partial buffer against the increase in late-night eating during the second sleep loss period. There is evidence that “banking” sleep can mitigate some of the neurobehavioral deficits resulting from sleep loss [37], and such banking may serve a similar purpose in regulating intake behaviors. Further research is needed to explore these possibilities. 

Although most intake variables did not differ during recovery from TSD or SR—as we had hypothesized—participants consumed a greater percentage of calories from protein on the first day of recovery from TSD, and tended to consume more calories on the first day of recovery from SR. These findings are in line with studies showing most [23,24,25,26,27,28,29,30], but not all [30,31] neurobehavioral responses recover similarly after TSD or SR. Intake differences following recovery sleep may be due to variations in sleep duration, timing, quality and architecture. Notably, these sleep parameters, which may differ when recovering after TSD versus SR, have been shown to modulate the percentage of caloric intake derived from protein as well as other energy balance measures [35,38,39,40,41,42,43]. Future studies are needed to explore this and other possible mechanisms underlying recovery intake differences between SR and TSD. 

As predicted, in men, late-night caloric intake was nearly 200 kcals higher and caloric intake the day following sleep loss was more than 600 kcals higher (with a 1.4% protein intake increase), across SR and TSD. Our results are in concordance with prior studies showing marked gender differences in energy intake with sleep loss [1,10,21]. In this study, daily caloric intake was also more stable in men, consistent with our prior findings [20]. A number of reasons may explain these gender differences, including differences between men and women in sex and metabolic hormones, peripheral controls of eating, or in eating behaviors and attitudes about food [10].

Our study had a few limitations. The participants were all healthy, and between the ages of 21 and 50 years old with BMIs in the normal to overweight range. As such, our results may not be generalizable to other groups, such as adolescents, obese individuals, or the elderly. In addition, we examined only healthy adult sleepers with habitual sleep durations of approximately 8 h per night. A recent study found habitually long sleepers had a higher sweet taste preference and decreased activity following sleep restriction, whereas habitually short sleepers did not experience such changes, suggesting the former group may be more vulnerable to energy balance changes following sleep loss [44]. Thus, future research should determine the stability of caloric and macronutrient intake, and late-night eating responses to sleep restriction and total sleep deprivation in normal weight, overweight and obese individuals of varying ages and with varying habitual sleep durations.

## 5. Conclusions 

Caloric and macronutrient intake during the late-night period of sleep loss and during the day following one night of SR and one night of TSD were consistent within individuals with differential vulnerability between individuals. Caloric intake did not differ during the late-night period of one night of SR and one night of TSD, although participants consumed a greater percentage of calories from protein and saturated fat during SR. Participants also consumed a greater percentage of calories from saturated fat during the day following SR than following TSD, and a greater percentage of calories from protein during recovery after TSD. Caloric intake was greater in men during the sleep loss period. We show, for the first time, robust differential vulnerability and phenotypic stability of energy balance responses to two commonly experienced types of sleep loss, heralding the use of biomarkers and countermeasures for prediction and mitigation of this critical vulnerability. These novel findings are timely, given worldwide increases in nighttime and overall caloric consumption, and in the prevalence of obesity and sleep loss.

## Figures and Tables

**Table 1:** Participant characteristics (Mean ± SD).

	*N*	Age (Years)	BMI (kg/m^2^)	Women	African American	Chronotype ^a^	Sleep Duration (h) ^b^	Sleep Midpoint (Time ± h) ^b^
All Participants	66	34.4 ± 9.0	24.4 ± 3.2	32 (48.5%)	48 (72.7%)	42.1 ± 5.9	8.0 ± 0.5	03:34 ± 0.8
Condition A (SR first)	34	33.2 ± 8.9	24.6 ± 3.0	16 (47.1%)	24 (70.6%)	40.9 ± 6.1	8.1 ± 0.4	03:41 ± 0.8
Condition B (TSD first)	32	35.6 ± 9.1	24.2 ± 3.3	16 (50.0%)	24 (75.0%)	43.6 ± 5.3	8.0 ± 0.6	03:27 ± 0.8

^a^ Morningness–Eveningness Composite Scale [33]; ^b^ Determined by wrist actigraphy (one week prior to study entry).

**Table 2:** Mean ± SD daily intake during the day following sleep restriction (SR) and the day following total sleep deprivation (TSD).

	Day Following SR (08:00–22:00; *N* = 66)	Day Following TSD (06:00–22:00; *N* = 66)	*p* Values
Kcal	2326.6 ± 811.1	2178.9 ± 793.7	0.11
Protein (%kcal)	12.4 ± 3.3	12.8 ± 3.4	0.50
Carbohydrate (%kcal)	58.4 ± 7.3	59.0 ± 8.6	0.56
Fat (%kcal)	31.2 ± 7.1	30.4 ± 8.1	0.31
Sugar (%kcal)	28.7 ± 7.5	28.4 ± 8.8	0.94
Saturated Fat (%kcal)	11.0 ± 3.5	10.0 ± 3.5	0.03
Fiber (g)	19.6 ± 9.6	18.5 ± 10.0	0.38

**Table 3:** Mean ± SD intake for each of the four days following recovery sleep (12 h TIB from 22:00–10:00, R1–R4, *N* = 24) from sleep restriction (SR; *N* = 12) or total sleep deprivation (TSD: *N* = 12).

	R1		R2		R3		R4	
	SR	TSD	SR	TSD	SR	TSD	SR	TSD
Kcal	2307.9 ± 615.0 ^†^	2007.4 ± 659.0	2055.3 ± 607.7	2060.1 ± 654.0	2096.2 ± 761.6	2018.7 ± 751.9	2026.6 ± 863.8	1861.2 ± 630.0
Protein (%kcal)	10.8 ± 3.3	16.2 ± 5.0 *	12.7 ± 4.8	17.6 ± 3.3	11.8 ± 3.8	16.2 ± 5.0	15.0 ± 7.8	15.1 ± 3.8
Carbohydrate (%kcal)	63.1 ± 6.0	55.2 ± 10.0	61.4 ± 10.2	52.7 ± 11.1	62.6 ± 8.6	57.8 ± 8.0	56.7 ± 13.0	53.0 ± 9.5
Sugar (%kcal)	31.2 ± 6.9	24.1 ± 9.6	29.0 ± 9.9	23.2 ± 4.6	34.9 ± 12.6	27.7 ± 7.2	26.8 ± 7.6	26.9 ± 6.6
Fat (%kcal)	28.1 ± 5.6	30.0 ± 7.6	27.8 ± 7.3	31.8 ± 8.8	27.4 ± 7.6	28.3 ± 7.7	31.7 ± 6.8	33.5 ± 7.6
Saturated Fat (%kcal)	10.4 ± 3.1	11.9 ± 3.4	10.1 ± 4.1	12.7 ± 4.5	8.5 ± 2.9	11.5 ± 4.4	10.7 ± 3.4	12.6 ± 4.2
Fiber (g)	20.7 ± 7.1	21.4 ± 9.3	17.7 ± 11.1	19.0 ± 10.4	12.0 ± 5.7	21.1 ± 15.5	15.1 ± 6.3	16.8 ± 9.2

^†^ *p* = 0.07: Participants tended to consume more calories on the first day of recovery (R1) from SR than from TSD; however, this did not reach statistical significance. * *p* < 0.001: Protein intake was significantly higher on the first day of recovery (R1) from TSD than from SR.
